# Necrotizing Infiltrative Lipomatosis in a Miniature Zebu Bull (*Bos primigenius indicus)*

**DOI:** 10.4061/2010/810496

**Published:** 2009-09-01

**Authors:** Scott D. Reed, Dawn E. Evans

**Affiliations:** Department of Pathobiological Sciences, School of Veterinary Medicine, Louisiana State University, Baton Rouge, LA 70803, USA

## Abstract

Lipomatosis is described in a miniature Zebu, *Bos primigenius indicus*, bull that died of perianesthetic complications. This is the first pathologic description of lipomatosis that we are aware of in this species and breed of cattle. Infiltration of multiple visceral organs is described and depicted along with comparison to previously published cases of lipomatosis in other breeds of cattle.

A 131 kg, three-year-old, miniature Zebu bull with a body condition score of five out of nine presented to the LSU SVM farm animal medicine service for repair of a tail fracture. During anesthetic induction the bull developed a ventricular tachyarrhythmia and subsequently succumbed. At necropsy, there were large amounts of hard nodular accumulations of fat surrounding and infiltrating many of the abdominal visceral organs and support structures. The mesentery surrounding the small intestines and colon was infiltrated with abundant firm fat that compressed intestines and filled greater than 40% of the peritoneal cavity. The retroperitoneal space was also distended with firm fat which was mottled white, tan, and golden-brown, and was often gritty when sectioned. Approximately 80% of the jejunum and 90% of the duodenum had diffuse transmural reddening, and the aborad half of the abomasum was edematous and reddened transmurally. Grossly, no other pathologically relevant lesions were detected. 

 Microscopic examination of peritoneal fat confirmed necrosis of large areas of fat (Figure 1). The perirenal and mesenteric lymph nodes revealed infiltration and partial effacement by adipose tissue. Normal lymph node architecture was lost and the remaining tissue was composed of widely separated lymphoid aggregates with interposed fat (Figure 2). The pancreas also had marked amounts of adipose tissue expanding the interstitium causing wide separation of islands of acinar tissue (Figure 3). Within abdominal fat there were multifocal areas of fat necrosis with lightly basophilic intracellular debris (mineralization). Other large foci of extracellular mineralization were also seen throughout the omental fat as characterized by large aggregates of intensely basophilic material. Sections of small intestines had a wide band of mature adipose tissue within the tunica muscularis, splitting the muscle into a thick inner circumferential layer and a thin longitudinal outer layer (Figure 4). Mature adipose tissue also frequently infiltrated the intestinal submucosa. In sections of duodenum taken from an area with a large mass of serosal fat, the muscle layers appeared compressed and attenuated. There were also multifocal infiltrates of mature adipose tissue within the esophageal muscle wall and throughout the thymic interstitium. 

 A definitive cause for ventricular fibrillation and sudden peri-anesthetic death could not be determined. No pathologically significant gross or histologic infiltration of the myocardium with fat was seen, nor was there evidence of primary cardiac disease or other definitive extracardiac disease known to cause ventricular fibrillation. It is unclear to what extent organ fat infiltration contributed to impairment of other organ function. 

 Lipomatosis is often an incidental finding but has been purported to cause intestinal obstruction and compression of other structures such as the ureters resulting in death [1–4]. Dystocia has also been reported from partial obstruction of the pelvic canal [5]. In this case the extramural compression of small intestines with transmural reddening appeared significant grossly; however, the degree of autolysis obscured our ability to detect any significant mucosal changes microscopically. 

 Pancreatic lipomatosis is thought to be capable of causing pressure atrophy of the pancreas but is not thought to be functionally significant [5]. The infiltrative pattern seen throughout the pancreatic interstitium in this case would suggest possible functional impairment, however there was no evidence of clinically significant pancreatic insufficiency nor was there evidence of clinically significant pancreatitis. 

 Description of fat infiltrating thoracic organs such as the esophagus and thymus, as was seen in this bull, has not been previously described. Most accounts of lipomatosis specifically describe abdominal involvement. Previous reports of extra-abdominal organ involvement are sparse and indicate that extra-abdominal involvement only occurred when the abdominal lipomatosis was advanced. Heart involvement has rarely been reported; Stuedemann et al. describe fat necrosis in adipose tissues adjacent to the heart and in tissues near the parotid salivary glands [3]. 

 Historically lipomatosis and fat necrosis have been used interchangeably in reference to the same syndrome in cattle [1–8]. The term lipomatosis arose because of the tumor-like gross appearance of the lesions (which are not neoplastic) [4]. In early reports of lipomatosis in cattle, lipomatosis was used to refer to a diffuse overgrowth of fatty tissue, either local or general [6]. The condition had also been described previously as diffuse lipoma of the abdominal cavity of cattle [6]. Other terms that have been applied to lipomatosis are sclerosing lipogranuloma, fat necrosis, hereditary multiple lipomatosis, diffuse lipomatosis, multiple lipomata, lipogranuloma, bovine fibrolipomatosis, bovine lipomatosis, primary lipogranuloma and primary lipogranulomatosis [5, 6, 8]. In this report, we have chosen to use the term lipomatosis because it is the most consistently used term, and we have used necrotizing and infiltrative to describe the specific attributes exhibited in this bull. 

 Abdominal fat necrosis and lipomatosis have been attributed to obesity, genetic predisposition, a diet rich in saturated fats or plant-derived lipases, a diet deficient in antioxidants, and consumption of tall fescue forage infected with the endophytic fungus *Acremonium coenophialum* [1, 3, 4, 6–10]. In rats, fat necrosis has been induced experimentally by feeding a diet rich in either long-chain saturated fatty acids or their esters [7]. 

 During fat necrosis in the rat, saponified fat cells undergo hydrolysis to fatty acids and glycerol. Glycerol is soluble and is carried away; however, unsaturated or short-chain fatty acids that are liquid at the temperature of the surrounding tissue dissolve less readily and bind basic ions. The resulting local acidity is thought to cause necrosis. Affected cells may eventually be replaced by fibrous tissue or may become calcified [7]. 

 Pigs fed diets high in unsaturated acids produce soft fat whereas diets low in fat cause hard fat. Up to 70% of hard fat is made up of glycerides of stearic, palmitic, and oleic acids. Because of the body's tendency to deposit fat similar to that which is ingested, some authors have suggested that fat necrosis is induced solely by feeding high levels of hard-fat-containing long-chain saturated fatty acids [7]. 

 Rabbits fed high levels of raw soybeans, a plant rich in plant lipase, developed high serum lipase levels and on necropsy had necrosis of perirenal fat which was prevented when soybeans were cooked. These studies concluded that plant lipase was absorbed intact and was responsible for the necrosis in rabbits. In swine however, fat necrosis could not be induced by feeding high levels of soybeans [7]. 

 Cases of fat necrosis have also been mentioned in association with selenium deficiency in horses, and nutritional myodegeneration in lambs with normal selenium but deficient vitamin E status [7]. 

 In cattle, lipomatosis and fat necrosis are more likely to be multifactorial, and in many cases may be complicated by feeding *Acremonium coenophialum* contaminated fescue. A similar pathogenesis has been suggested in Elds Deer, *Cervus eldi thamin *[10]. In the early 1960s it was recognized that cows grazing chicken broiler-littered fescue (BLF) pasture had an average body temperature above normal and higher than those of cows grazing other fescue pastures. Cattle grazing BLF sought methods of lowering heat stress (standing in water, seeking shade) and had above normal respiration rates compared to cattle grazing moderately or low ammonium nitrate-fertilized fescue. When stressed (vaccinating, herding), the cows grazing BLF appeared to experience greater stress, which partly could have resulted from overall body condition, but was also considered consistent with fescue toxicity [4, 8]. Williams et al. further suggested that the effect of pyrexia on fat caused it to undergo transformation to a form that crystallizes and is treated as a foreign body by the body. Toxic influences on hepatocytes could also alter fat metabolism sufficiently to result in greater production of saturated fatty acids likely to undergo crystallization [4]. 

 In the 1980s BLF-grazing cattle with signs of summer fescue toxicosis were found to have low cholesterol, low body condition, low coat scores, increased respiratory rate, increased heart rate, and excess salivation, and had the highest degree of endophytic fungus infestation in their fescue pasture. BLF-grazing cattle also had high levels of N-acetyl and N-formyl loline which are fungal metabolites thought to influence lipid metabolism in cattle (and may have influenced fat necrosis and summer fescue toxicosis) [3]. In the bull reported herein, the diet varied from a variety of unspecified grains, grass and hay. 

 The degree of fat infiltration in multiple organs including lymph nodes, thymus, esophagus, pancreas, and intestines was marked and may have caused some functional impairment in this bull. Intestinal infiltration and pancreatic infiltration have been described or depicted previously [4, 6, 8], but we are unaware of previous reports of esophageal, lymph node, or thymus infiltration as was seen in this bull. 

 The spectrum and degree of fat necrosis seen in this case are consistent with previously described cases in other breeds of cattle and deer [1–10]. The lack of significant inflammatory infiltrate and limited areas of necrosis are commonly reported and are consistent with the proposed noninflammatory pathogenesis of lipomatosis and fat necrosis in cattle and deer [4, 6, 8–10]. 

 In summary, the bull reported here is the first case we are aware of reported for lipomatosis in a miniature Zebu bull. Both previously reported mass effects and fat necrosis were seen in this case, along with extensive infiltrative behavior that has been heretofore unreported. The role of diet and fungal toxins in this case is not determined. 

## Figures and Tables

**Figure 1:**
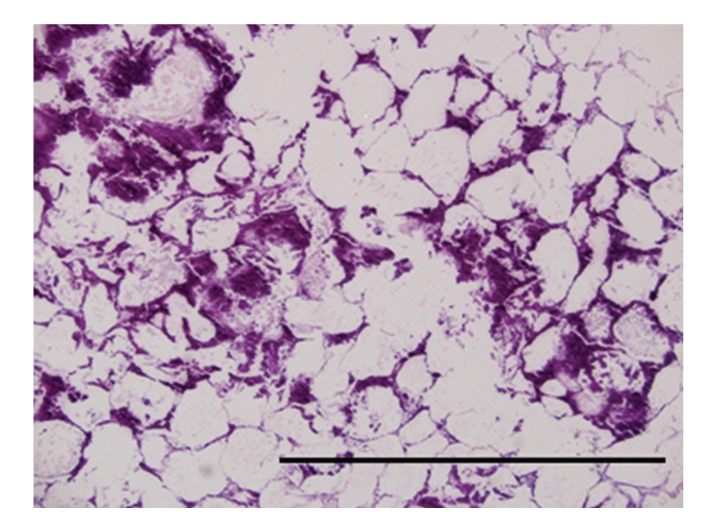
Photomicrograph of an area of fat necrosis and mineralization. Hematoxylin and eosin stain. Bar = 1 mm.

**Figure 2:**
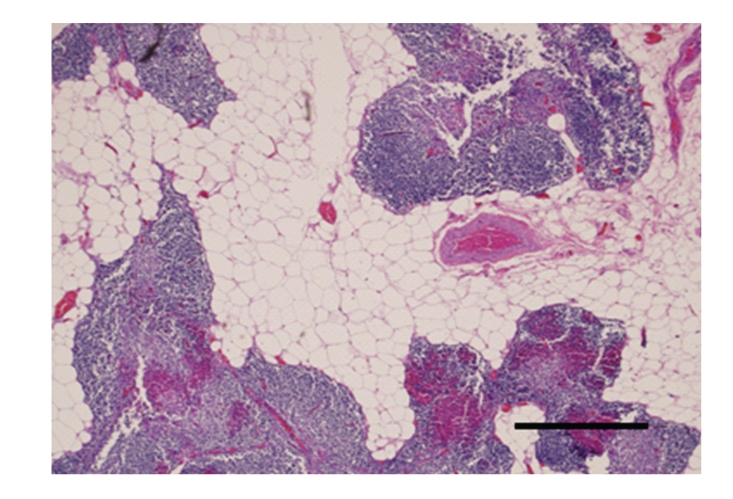
Normal lymph node architecture was lost and the remaining tissue was composed of widely separated lymphoid aggregates with interposed fat. Hematoxylin and eosin stain. Bar = 1 mm.

**Figure 3:**
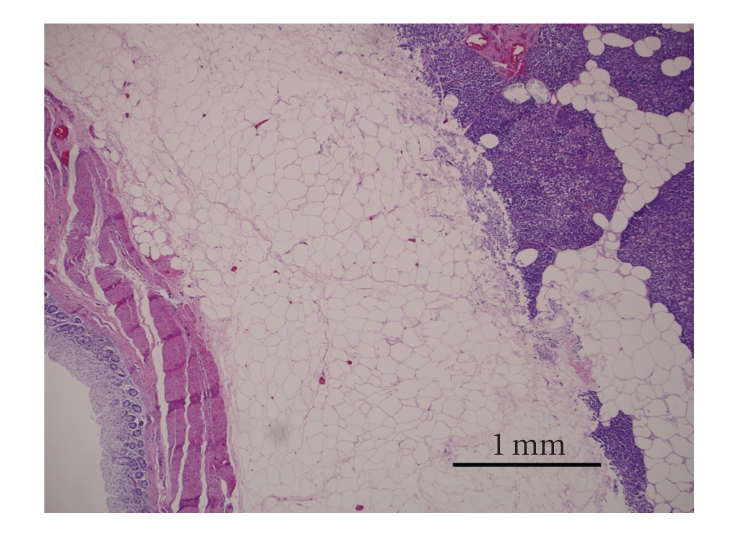
The pancreas also had marked amounts of adipose tissue expanding the interstitium causing wide separation of islands of acinar tissue. Hematoxylin and eosin stain. Bar = 1 mm.

**Figure 4:**
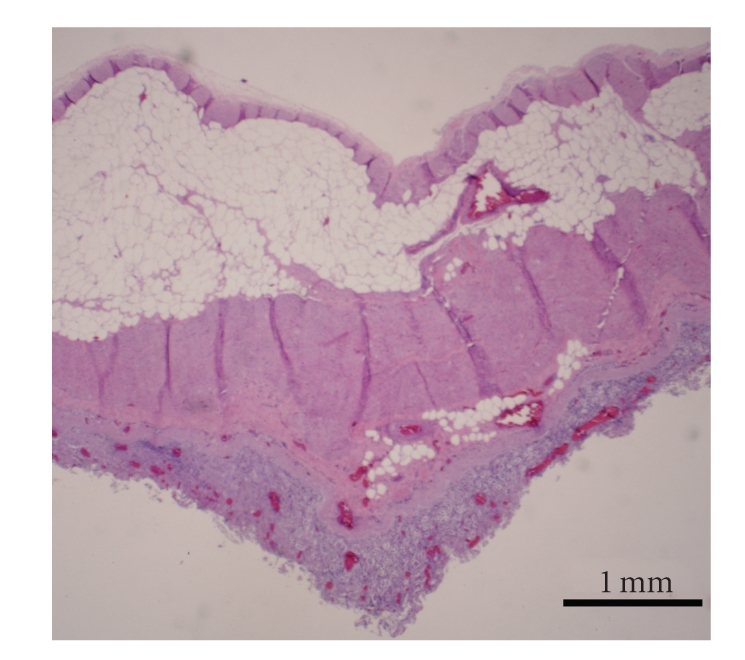
Sections of small intestines had a wide band of mature adipose tissue within the tunica muscularis, splitting the muscle into a thick inner circumferential layer and a thin longitudinal outer layer. Hematoxylin and eosin stain. Bar = 1 mm.
